# Synthetic biology for heparin biomanufacturing

**DOI:** 10.1186/s40643-025-00999-x

**Published:** 2026-01-27

**Authors:** Yingjia Pan, Zhangliang Liu, Peng Wang, Jian Gao, Junzhong Li, Binying Lv, Junyue Li, Xiangjiu Zhang, Liang Yin, Chang Dong, Jiaying Wang, Lei Huang, Jiazhang Lian, Jine Li, Jinshan Li, Zhinan Xu

**Affiliations:** 1 Key Laboratory of Biomass Chemical Engineering of Ministry of Education, College of Chemical and Biological Engineering, Zhejiang University, Hangzhou, 310027 China; 2 ZJU-Hangzhou Global Scientific and Technological Innovation Center, Zhejiang University, Hangzhou, 310000 China; 3 State Key Laboratory of Microbial Diversity and Innovative Utilization, Institute of Microbiology, Chinese Academy of Sciences, Beijing, 100101 China; 4 College of Life Sciences, University of Chinese Academy of Sciences, Beijing, 100049 China; 5 Tianjin Institute of Industrial Biotechnology, Chinese Academy of Sciences, Tianjin, 300308 China

**Keywords:** Bioengineered heparin, Heparosan fermentation, Chemoenzymatic modification, De novo biosynthesis, Synthetic biology

## Abstract

Heparin, mainly used as an anticoagulant, has also shown potential in the treatment of diseases such as inflammation and cancer. Currently, heparin is mainly extracted from the intestinal mucosa of pigs. However, due to concerns about disease transmission and contamination associated with animal-derived products, biomanufacturing techniques have been explored as alternative production methods. Through enzyme engineering, metabolic engineering, and synthetic biology approaches, the heparin biosynthetic pathways have been systematically optimized. The main biomanufacturing techniques include in vivo/in vitro combination strategy (microbial heparosan fermentation followed by chemoenzymatic modification) and de novo biosynthesis. This article comprehensively discusses the latest advancements, challenges, and future perspectives of these heparin biomanufacturing techniques.

**Graphical abstract:**

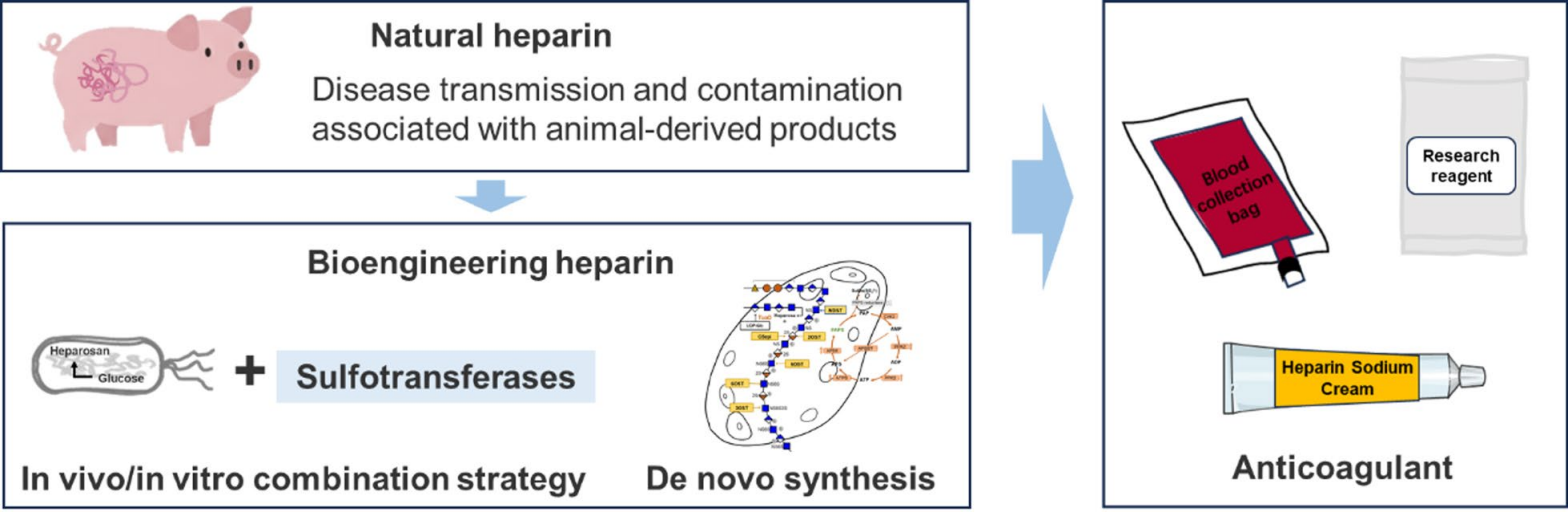

## Introduction

Heparin is a naturally occurring, highly sulfated glycosaminoglycan (GAG) widely used as an anticoagulant in clinical settings (Linhardt 2003). It plays a critical role in the prevention and treatment of thrombotic disorders such as deep vein thrombosis, pulmonary embolism, and myocardial infarction, as well as in medical procedures including dialysis and cardiopulmonary bypass. Heparin is produced by mast cells and basophils in mammals and remains a critical medication listed by the World Health Organization as essential (Baytas and Linhardt 2020; Thacker et al. 2022).

Natural heparin is a highly sulfated subtype of heparan sulfate (HS), primarily produced by mast cells, possessing strong anticoagulant activity, whereas HS is ubiquitously present on all mammalian cell surfaces and has diverse functions. Heparin’s anticoagulant mechanism is based on its ability to bind to and activate antithrombin III, a natural inhibitor of several key enzymes in the coagulation cascade, especially thrombin (factor IIa) and factor Xa. The unique pentasaccharide sequence within heparin activates antithrombin and inhibits factor Xa. Additionally, long-chain heparin can bind both antithrombin and thrombin to form a ternary complex, effectively inhibiting the activity of factor IIa. This mechanism forms the basis for heparin’s widespread use as an anticoagulant in various clinical settings (Gray et al. 2012). Based on molecular size, heparin is classified into unfractionated heparin (UFH), low molecular weight heparin (LMWH), and ultra-low molecular weight heparin (ULMWH). UFH consists of heterogeneous polysaccharide chains (3–30 kDa) with potent but variable anticoagulant activity. LMWHs are depolymerized derivatives of UFH with smaller chain lengths (~ 1–10 kDa), offering more predictable pharmacokinetics and a primarily anti-factor Xa effect, allowing subcutaneous administration without routine laboratory monitoring. ULMWHs typically have a molecular weight below 3 kDa. They are synthetic or derived by further depolymerizing LMWHs, resulting in a more uniform molecular structure and more stable anticoagulant effects (Yang et al. 2022; Zhang et al. 2025).

**Fig. 1:**
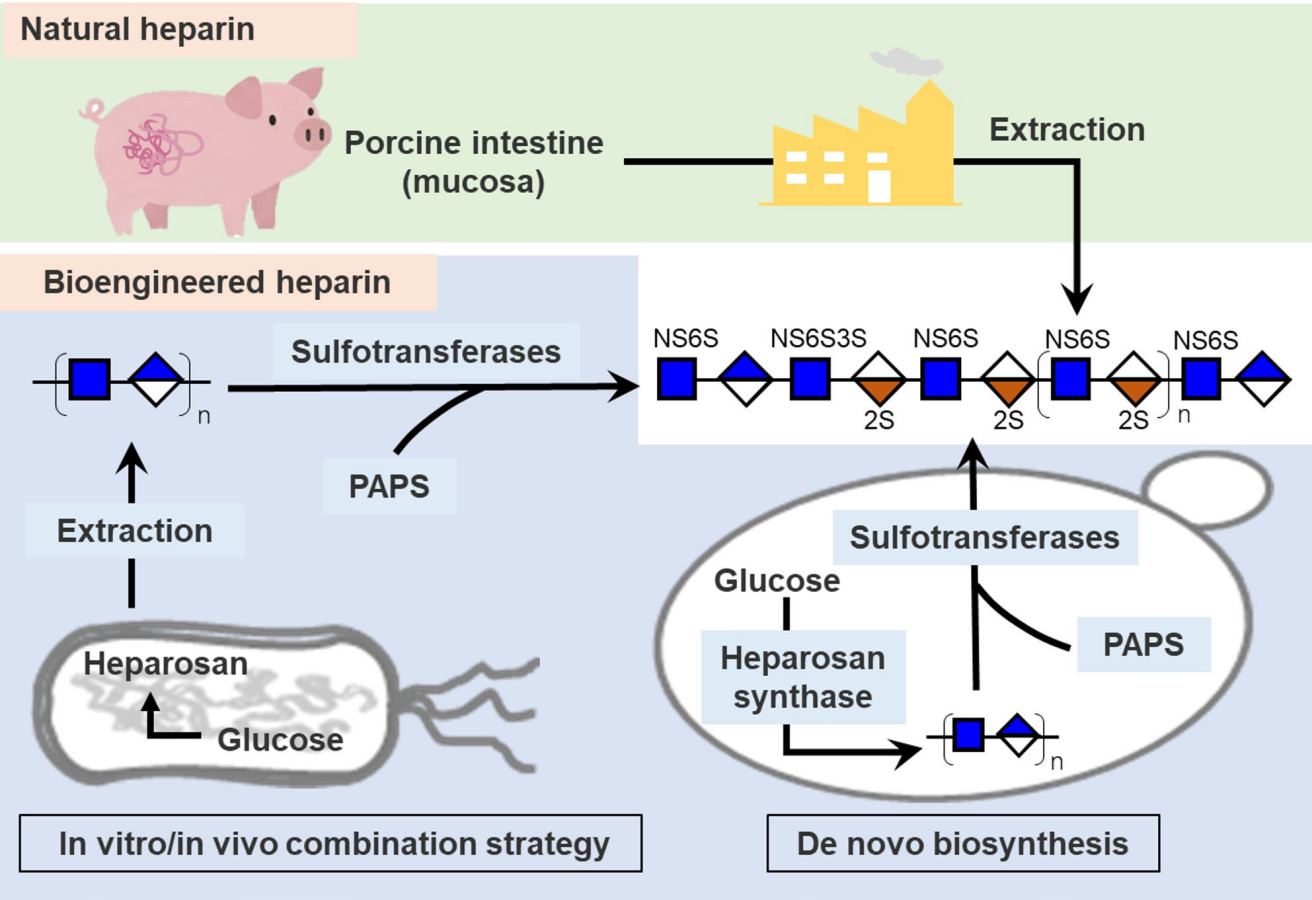
Schematic diagram of different heparin production strategies. Natural heparin is produced by extraction from animal tissues (e.g., porcine intestine), while bioengineered heparins can be produced by 1) in vitro/in vivo combination strategy, which starts with heparosan extracted from prokaryotic organisms, followed by enzymatic modification; or 2) de novo biosynthesis, in which heparin is directly synthesized from simple carbon sources within eukaryotic cells

Heparin production encompasses multiple methodologies, each with distinct advantages and challenges (Dulaney and Huang 2012; Douaisi et al. 2024; Baytas and Linhardt 2020; Deng et al. 2024). Currently, heparin is primarily extracted from porcine intestinal mucosa, with the majority of raw material pigs concentrated in a few countries, notably China. However, since 2018, the outbreak of African swine fever has led to a significant reduction in China’s pig population, with approximately 500 million pigs lost by 2019—equivalent to a decrease of over 80 tons in heparin production capacity—resulting in global supply shortages. The subsequent COVID-19 pandemic further disrupted supply chains, causing a short-term increase of 20–35% in raw material prices. With the global demand for heparin steadily rising and the market projected to reach approximately USD 62.1 billion by 2025, the overreliance on animal-derived sources has exposed considerable vulnerabilities in terms of supply stability, cost control, and production safety. Therefore, innovative production approaches such as biomanufacturing are urgently needed to enhance supply chain resilience and ensure the secure and sustainable availability of this critical medication (Guerrini et al. 2008)(Vilanova et al. 2019). Total chemical synthesis offers precise control over heparin oligosaccharide structure, although it involves complex multistep processes with low overall yield (Capila and Linhardt 2002). Chemoenzymatic synthesis, a promising alternative, mimics heparin’s natural biosynthesis by combining chemical modifications and enzymatic steps, producing bioengineered heparin with properties similar to pharmaceutical heparin, while enabling scalable and animal-free production (Xu et al. 2011). Chemoenzymatic bioengineered heparin has been produced at over 10 g scale, closely matching United States Pharmacopeia (USP) porcine heparin and enoxaparin in composition and activity (Douaisi et al. 2024). It is proposed as an animal-free clinical drug candidate. Recent advances also include microbial and metabolically engineered systems that biosynthesize heparin from simple carbon sources (such as glucose and glycerol) directly. These innovative methods aim to ensure safer, more consistent, and sustainable heparin supply, expanding therapeutic possibilities beyond traditional sources (Li 2016; Zhang et al. 2022). This article provides a comprehensive review of the production methods for bioengineered heparin, including in vivo/in vitro combination strategy (heparosan fermentation followed by chemoenzymatic modification) and de novo biosynthesis (Fig. 1). Additionally, the latest advancements, key technologies, and optimization strategies of these methods are highlighted. Finally, the challenges and future perspectives of bioengineered heparin are discussed.

## Microbial biosynthesis of heparosan

Following the recent elucidation of the biosynthetic pathway of native heparosan, the long-standing extraction paradigm reliant on porcine small intestinal mucosa and bovine lung tissue has been subjected to re-evaluation. Since the mid-twentieth century, pharmacopoeial-grade heparin has predominantly been obtained through extraction of crude material from animal tissues, followed by chemical depolymerization and fractionated purification. However, this route is constrained by batch-to-batch variability in raw materials, risks of pathogen transmission, supply chain volatility, and limited control over structural uniformity (Bhaskar et al. 2012). Microbial biosynthesis has emerged as an efficient and scalable route for heparin production owing to rapid advances in synthetic biology. Identification of native heparosan-producing strains has established the basis for an animal-free, scalable, and programmable two-stage platform that integrates heparosan fermentation with in vitro chemoenzymatic modification.

### Native microbial heparosan biosynthetic pathway

**Fig. 2:**
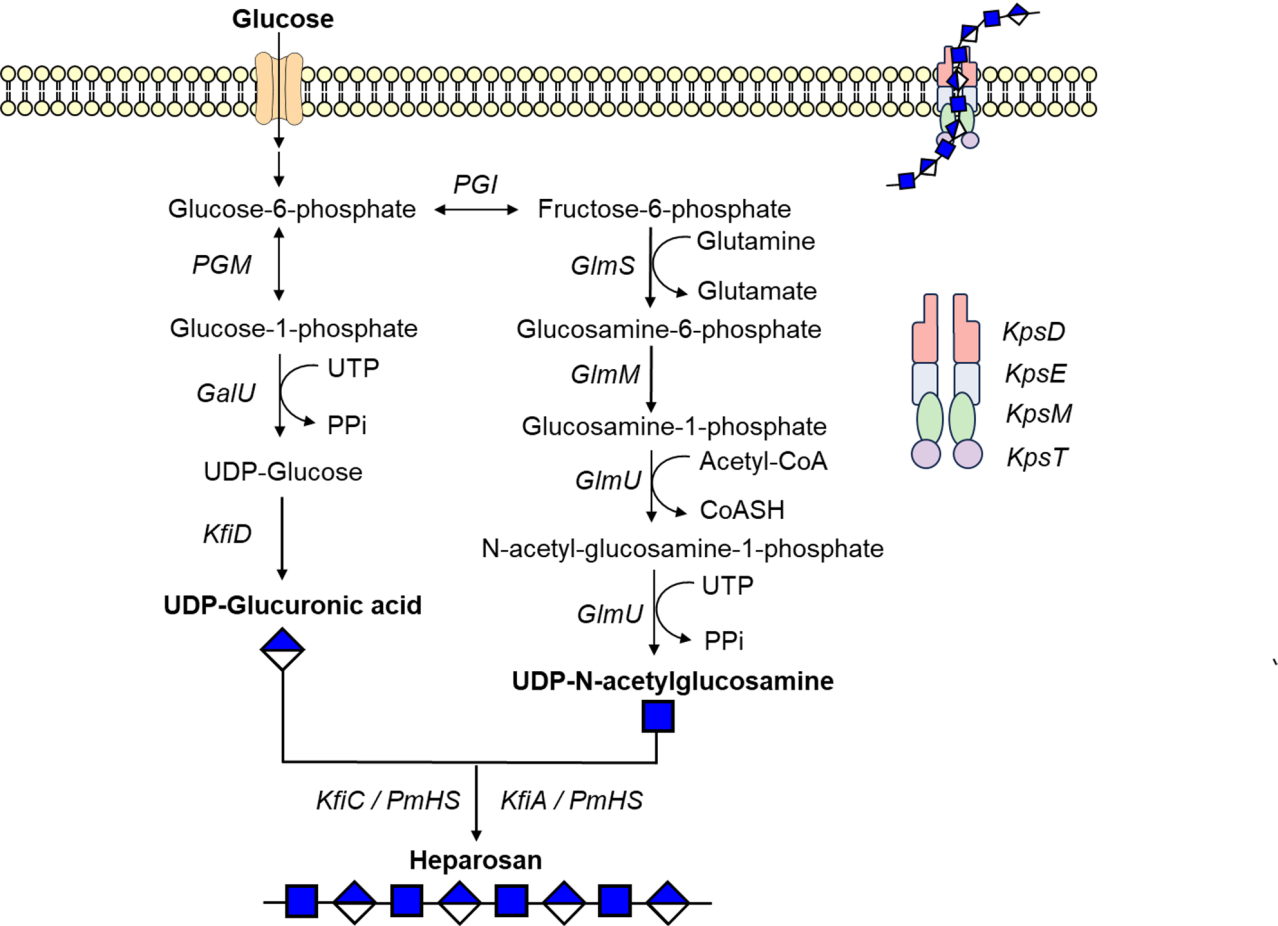
Schematic diagram of microbial biosynthesis of heparosan

Heparosan is a linear GAG characterized by alternating α-1,4- and β-1,4-glycosidic bonds that connect glucuronic acid (GlcA) and *N*-acetylglucosamine (GlcNAc) residues (Bhaskar et al. 2012). To date, heparosan has been isolated from four native microbial producers—*Escherichia coli* K5, *E. coli* Nissle 1917 (EcN), *Pasteurella multocida* type D, and *Avibacterium paragallinarum*—all of which possess complete heparosan biosynthetic pathways (DeAngelis et al. 2002; Vann et al. 1981; Wu et al. 2010; Shao et al. 2025). Central carbon flux is funneled into the formation of UDP-*N*-acetylglucosamine (UDP-GlcNAc) and UDP-glucuronic acid (UDP-GlcA), two essential UDP-sugar precursors for heparosan biosynthesis (Fig. 2). In the UDP-GlcNAc biosynthetic pathway, the glutamine-dependent amidotransferase GlmS catalyzes the first committed step, converting D-fructose-6-phosphate and L-glutamine to D-glucosamine-6-phosphate and L-glutamate. Phosphoglucosamine mutase (GlmM) converts glucosamine-6-phosphate to glucosamine-1-phosphate, after which the bifunctional enzyme GlmU sequentially acetylates it to *N*-acetylglucosamine-1-phosphate and then catalyzes the uridyltransferase (pyrophosphorylase) reaction to yield UDP-GlcNAc (Wang et al. 2020a). In the UDP-GlcA biosynthetic pathway, phosphoglucomutase (Pgm) catalyzes the reversible conversion of D-glucose-6-phosphate to D-glucose-1-phosphate, providing the substrate for UDP-glucose formation. UDP-glucose pyrophosphorylase (GalU) converts glucose-1-phosphate and UTP to UDP-glucose (UDP-Glc), which is subsequently oxidized by UDP-glucose dehydrogenase (KfiD) in two NAD^+^-dependent steps to yield UDP-GlcA.

In *E. coli* K5, a GlcNAc residue is first transferred from UDP-GlcNAc to the nonreducing terminus of the nascent heparosan chain by KfiA (an α1 → 4 *N*-acetylglucosaminyltransferase). Subsequently, a GlcA residue is transferred from UDP-GlcA by KfiC (a β1 → 4 glucuronyltransferase), and a strictly alternating [→ 4)-β-D-GlcA-(1 → 4)-α-D-GlcNAc-(1 →]ₙ backbone is thereby generated through iterative cycles. Stabilization of the KfiA–KfiC complex is provided by the accessory (chaperone-like) protein KfiB, whereby processivity is enhanced and a more uniform chain-length distribution is achieved (Hodson et al. 2000). In contrast, in *P. multocida*, alternating transfers of GlcA and GlcNAc from UDP-GlcA and UDP-GlcNAc, respectively, are catalyzed by a single bifunctional heparosan synthase (PmHS), and the requirement for separate monospecific glycosyltransferases and an accessory stabilizing factor is thus obviated (Otto et al. 2012). In EcN, KfiB is not directly involved in heparosan chain polymerization; instead, interactions with the capsule export ABC transporter components KpsM (inner-membrane permease) and KpsT (ATP-binding subunit) are exhibited, indicating an accessory role in coordinating or facilitating heparosan translocation (Hu et al. 2024).

After the heparosan chain is synthesized on the cytoplasmic face of the inner membrane, it is exported to the cell surface, where it forms the capsular polysaccharide layer. A conserved linker region comprising two 3-deoxy-D-manno-oct-2-ulosonic acid (Kdo) residues is first synthesized on the cytoplasmic face of the inner membrane by the Kdo transferases KpsC and KpsS. The ABC transporter complex, composed of KpsM (the multispanning inner membrane channel) and KpsT (the cytosolic ATP-binding subunit), then energizes translocation of the nascent capsular polysaccharide across the inner membrane into a periplasmic translocation conduit. The conduit formed by the periplasmic scaffold KpsE and the outer-membrane component KpsD is responsible for guiding the polymer through the periplasm and mediating its surface display as the capsule layer (Whitfield et al. 2020).

### Engineering microbial cell factories for heparosan production

Heparosan biosynthesis has evolved from a singular focus on high titer to a multi-objective strategy balancing titer, extracellular secretion, controllable molecular weight, safety, and regulatory compliance (Table 1). The move from native producers to safer chassis has simultaneously strengthened biosafety and simplified downstream processing. Current metabolic engineering emphasizes: (i) pathway reprogramming for flux redirection, (ii) precise tuning of UDP-sugar precursor ratios, (iii) synchronized polymerization with transport/export, and (iv) modular polymerase architectures (multi-enzyme assemblies, bifunctional catalysts, hierarchical chain-length gating), collectively producing a structured library of high-MW, low-MW, and oligomeric backbones for downstream sulfation customization.

**Table 1:** Strategies for microbial synthesis of heparosan

Host strain	Engineering strategies	Medium	Culture condition	Titer (g/L)	Molecular weight (kDa)	References
*E. coli* K5	Native host	Glucose + Glycerol	12 L fermenter	10.2	–	Viskov et al. 2008
*E. coli* K5	Optimizing fermentation process	M9/LB/Glucose/ Glycerol Glucose	Shake flask 7 L fermenter	0.07–0.5 15	82/68/44/79 84	Wang et al. 2010
*E. coli* K5	Optimizing fermentation process	Glycerol	3 L fermenter	8.63	–	Liu et al. 2012
*E. coli* K12	Co-expressing *kfiA, kfiB, kfiC, kfiD,* and *elmA* from *E. coli* K5	Glucose + Glycerol	0.5/3 L fermenter	1	DP 2–10 OS	Barreteau et al. 2012
*E. coli* BL21	Overexpressing *PmHS2* from *P. multicida*	5 mM UDP-sugar	/	/	~ 103	Chavaroche et al. 2010
*E. coli* BL21	Co-expressing *kfiA, kfiB*, *kfiC*, *kfiD*, and optimizing fermentation process	Glycerol	Shake flask 3 L fermenter	0.33 1.88	39.6–118	Zhang et al. 2012
*E. coli* BL21	Co-expressing *kfiA and kfiC* from *E. coli* K5	Glucose + Glycerol	0.5 L fermenter	1.5	–	Leroux and Priem 2016
*E. coli* BL21	Co-expressing *kfiA* and *kfiC* from *E. coli* K5	Glycerol	Shake flask 6 L fermenter	~ 0.28 ~ 0.48	~ 5–150	Roy et al. 2021
*B. subtilis* 168	Co-expressing *kfiA* and *kfiC* from *E. coli* K5; Overexpressing* tuaD*	Sucrose	Shake flask 3 L fermenter	2.65 5.82	67.7	Jin et al. 2016
*B. subtilis* 168	Overexpressing *PmHS1* from *P. multicide*; UDP-precursors pathway genes *tuaD*, *gtaB*, and *gcaD*	Glucose	Shake flask	0.24	39–53	Chen et al. 2017
*B. megaterium* MS941	Using a T7 expression system to express *PmHS2* from* P. multicida*	Glucose	Shake flask 1.5 L fermenter	0.25 2.74	10–40 200–300	Williams et al. 2019
*B. megaterium* DSM319	Co-overexpressing *kfiA* and *kfiC* from *E. coli* K5; Overexpressing UDP-precursors pathway genes *tuaD*, *gtaB*, *gcaD*, and *glmM*	Sucrose	3L fermenter	1.32	31–60	Nehru et al. 2020
*B. megaterium* DSM319	Co-overexpressing *kfiA* and *kfiC from E. coli* K5; Overexpressing *tuaD* and *gtaB*	Sucrose	Shake flask 3 L fermenter	0.20 1.96	41.9	Nehru et al. 2021
*B. megaterium*	Using the double Andrew’s model; Adding GlcA/GlcNAc to the culture medium	Sucrose	Shake flask 2.7 L fermenter	0.35 0.9	–	Nehru et al. 2024
*Synechococcus* PCC 7942	Co-overexpressing *galU* (from *E. coli* MG1655) and *PmHS2* (from* P. multicide)*	Photoautotrophic production	Shake flask	0.0028	–	Sarnaik et al. 2019
*C. glutamicum*	Co-overexpressing *kfiA* and *kfiC* from EcN; Overexpressing *galU*, *ugd*, *glmS*, *glmM*, and *glmU*; Biotin addition during the fermentation process; Membrane shielding strategy	Corn steep powder Glucose	Shake flask 3 L fermenter	0.1 5.8	2400	Hu et al. 2022a
*C.glutamicum* ATCC 13032	Overexpressing *PmHS2* from *P. multicide*; Overexpressing *ugdA*, *galU*, *whcD*, and *pnkB*	Glucose	Shake flask 5 L fermenter	1.4 7.02	801	Yu et al. 2025
*L. lactis* (SH6)	Overexpressing *kfiA*, *kfiC*, *ugd*, *glmU*,* and pgmA*	Glucose	3 L fermenter	1.26	10–20	Guhan et al. 2022
*E. coli* Nissle 1917 (EcN)	High-density fermentation	Glucose	100 L fermenter	3	68	Datta et al. 2020
EcN	Knockout *trxB*, *tetA*, and *gor*	Glucose	5 L fermenter	0.85	–	Li et al., 2021
EcN	Overexpressing ec*kfiD*, bs*galU*, ec*glmM*, *kfiA*, and *kfiC*	Glucose	Shake flask 3 L fermenter	0.8 11.5	625	Hu et al. 2022b
EcN	Amplifying the 19-kb *kps* locus; Overexpressing *kfiA* and *kfiC* from *E. coli* K5	Glucose	Shake flask 5 L fermenter	0.104 /OD 9.1	–	Yu et al. 2023
EcN	Optimizing RBS for *kfiACB* expression; Overexpressing *kpsTME*	Glucose	Shake flask 5 L fermenter	1.03 12.2	–	Hu et al. 2024
EcN	Knocking out *zwf*, *pfkAB*, *pgi*, and *fruA*; Overexpressing *glmM*, *pgm*, and *galU*	Glucose + Fructose + Glycerol	Shake flask	1.05	–	Shao et al., 2025
EcN	Overexpressing *galU*, *kfiD*, and *glmM*; Overexpressing *sacA* (from *B. subtilis*) or *spI* (from *Bifidobacterium adolescentis*)	Sucrose	Shake flask	0.62	–	Chen et al. 2025
EcN	Knockout *zwf* and *pfkAB*; Overexpressing* kfiD*	Glucose	Shake flask 5 L fermenter	1.04 4.34	–	Shao et al., 2025

#### Pathway reprogramming for flux redirection

Through process optimization or genetic modification, it is possible to redirect the carbon flow of the microbial central metabolic pathway to the synthesis of heparin precursors. High-cell-density fermentation of *E. coli* K5 was performed to achieve a heparosan titer of 8–15 g/L (Wang et al. 2010; Liu et al. 2012). Co-expression of *kfiA/B/C/D* and *PmHS2* in *E. coli* BL21, supported by chaperon-assisted folding, enhanced soluble synthase levels and delivered 1.5 g/L heparosan with a stabilized chain-length distribution (Leroux and Priem 2016). In *Corynebacterium glutamicum*, a multi-gene strategy (*kfiAC* plus *galU*, *ugd*, *glmS*, *glmM*, and *glmU*) combined with a membrane shielding approach yielded 0.10–5.80 g/L heparosan (Hu et al. 2022a). Subsequent integration of *PmHS2* with PorB-mediated membrane display, deletion of endogenous glycosyltransferases, and overexpression of the regulatory factor genes *WhcD* and *PnkB* further increased titers to 1.40–7.02 g/L (Yu et al. 2025).

#### Precise tuning of UDP-sugar precursor ratios

UDP-GlcA and UDP-GlcNAc are the key precursors for the synthesis of heparosan. By enhancing the expression of precursor synthesis genes and balancing the supply ratio of the two precursors, this approach can avoid limitations in synthesis efficiency caused by the insufficiency of a single precursor. Hu et al. reported that co-overexpressing UDP-sugar precursor pathway genes (*galU* and *glmM*) together with the heparosan polymerase increased the heparosan titer to 11.50 g/L and substantially elevated the production molecular weight (Hu et al. 2022b). In *B. megaterium*, dual promoter expression of *kfiA* and *kfiC*, followed by overexpression of *tuaD* and *gtaB*, increased the titer to 1.96 g/L with an average molecular weight of 41.90 kDa (Nehru et al. 2020). Similarly, *Lactococcus lactis* expressing *kfiAC* with enhanced *ugd*, *glmU*, and *pgm* expression resulted in 1.26 g/L heparosan (Guhan et al. 2022).

#### Synchronized polymerization with transport/export

During heparin precursor biosynthesis, extracellular secretion of the heparosan polymer was enhanced, whereby feedback inhibition between the accumulated intracellular polymer and sugar nucleotide donors was alleviated, cellular metabolic and energy homeostasis was stabilized, product titer was increased, and polymer chain length and molecular weight distribution were improved. Targeted chromosomal amplification of a ~ 19 kb heparosan biosynthesis–export locus (encompassing polymerase and *kps* transport genes) increased heparosan production to 9.10 g/L (Yu et al. 2023). *PmHS2* expression levels and membrane-anchored localization were fine-tuned to shorten the diffusion distance between the polymer synthesis sites and the putative export interface, thereby decreasing intracellular retention of the free high-viscosity polymer (Hu et al. 2022b; Williams et al. 2019). In *C. glutamicum*, hydrophobic transmembrane segments or secretory signal peptides were employed to semi-anchor the synthase at the perimembranous region, thereby advancing the polymerization interface toward the inner cell envelope and indirectly facilitating polymer export (Zhao et al. 2025). Regulation or engineering of Gram-negative outer membrane porins was employed to enhance the diffusion of polysaccharides from the periplasm to the extracellular milieu; this approach has been successfully applied to hyaluronan biosynthesis and was considered potentially effective for heparosan biosynthesis as well (Liu et al. 2023b; Wang et al. 2020b). Meanwhile, intracellular polymerization and limited extracellular secretion were associated with the accumulation of products within the cytoplasm or periplasm, which necessitated downstream cell lysis and nucleic acid/host cell protein (HCP) removal. As a result, the complexity of clarification and initial purification was substantially increased. Compared with intracellular products that required lysis followed by precipitation or nuclease treatment, secreted polysaccharides in the supernatant were rapidly clarified and concentrated using a sequence of continuous centrifugation, depth filtration, and ultrafiltration or alcohol precipitation, thereby shortening the process and reducing variable costs (Shi 2016). For hosts at risk of endotoxin exposure (e.g., *E. coli)*, the release of lipopolysaccharide (LPS) during cell lysis was reduced by extracellular secretion, thereby facilitating endotoxin removal. In secretion-dominant Gram-positive hosts (e.g., *B. subtilis*), the target polysaccharide/protein was directly introduced into the culture supernatant, and after clarification, it was initially purified by ultrafiltration or by one- or two-step capture(Liu and Yu 2025). In Gram-negative hosts such as *E. coli*, products that had accumulated in the intracellular or periplasmic space were typically subjected to high-pressure homogenization or chemical cleavage, which resulted in a significant release of LPS endotoxins. Consequently, additional purification methods were introduced downstream, increasing the difficulty and cost of separation (Pouresmaeil and Azizi-Dargahlou 2023). In the engineered production of heparosan, hosts with stronger secretory capacity and thinner cell wall barriers were selected, and the polymerization and transport systems were coupled, thereby reducing the risk of co-extraction with intracellular macromolecules and decreasing the difficulty of downstream separation from a design perspective (Put et al. 2024).

#### Modular polymerase architectures

The composition and functionality of the heparosan polymerase system have been refined through advanced metabolic engineering and synthetic biology strategies. Specifically, multi-enzyme complexes, bifunctional enzymes, and regulated enzyme expression for chain length control was employed. As a result, polymerization efficiency, molecular weight controllability, and product homogeneity were significantly improved. Heparosan biosynthesis and partial control of polymer chain length were achieved in non-pathogenic *E. coli* K12 and BL21 by heterologous expression of *kfiA*, *kfiB*, *kfiC*, *kfiD* together with *elmA* (Zhang et al. 2012; Roy et al. 2021; Leroux and Priem 2016; Barreteau et al. 2012). In *B. subtilis*, expression of *PmHS1* was employed to replace conventional single-function glycosyltransferases (such as KfiA and KfiC), thereby simplifying the polymerization system. Additionally, precursor enhancement pathway genes were integrated to further promote product biosynthesis (Chen et al. 2017). In *B. subtilis*, expression of *PmHS1* with augmented UDP precursor pathways yielded 0.238 g/L heparosan (39–53 kDa) (Jin et al. 2016). These modular designs facilitate precise control over polymerization and product characteristics. The maturation of intelligent heparosan biomanufacturing is anticipated to be further enabled by integrating emerging technologies, such as dynamic adaptive regulation and machine learning–based predictive modeling.

#### Strategies for controllable molecular weight synthesis

Different molecular weights of heparosan are key factors that determine the clinical applications, anticoagulant intensity and safety of finished heparin products (Hemker et al. 2019; Javot et al. 2009). Thus, achieving controlled synthesis of heparosan with specific molecular weights is a core prerequisite for the precise preparation of clinically applicable heparin drugs. Currently, strategies to regulate the microbial synthesis of heparosan with varying molecular weights mainly focus on three aspects: substrate supply, key enzyme screening, and expression regulation during glycosidic chain elongation.

Heparosan synthesis relies on two substrates, UDP-GlcA and UDP-GlcNAc. Supplying different ratios of these UDP-sugars to the heparosan synthesis system provides corresponding substrate support for its production. Based on this, the molecular weight of heparosan can be directionally regulated by controlling and optimizing the UDP-sugar synthesis pathway. For instance, Zhang et al. enhanced UDP-sugar supply by co-overexpressing *ugd* and *glmS* in the synthesis pathway, which increased the molecular weight of heparosan from 2500 to 3600 kDa (Hu et al. 2022a). Further studies showed that simply increasing UDP-GlcA supply significantly raised the molecular weight of synthesized heparosan, while raising UDP-GlcNAc supply from 25 μM to 5 mM reduced the molecular weight of the products (Nehru et al. 2020; Lidholt et al. 1988; Chen et al. 2017).

The glycosidic chain elongation of heparosan depends on the glycosyltransferases KfiA and KfiC. The expression levels and activities of these two enzymes directly determine the rate and length of glycosidic chain elongation, and thus affect the molecular weight of heparosan. Therefore, the molecular weight of heparosan can be effectively regulated by controlling the expression of these key enzymes. Hu et al. used ribosome-binding site (RBS) engineering to adjust the translation rates of KfiA and KfiC, which successfully increased the molecular weight of heparosan from 312.39 kDa to 624.52 kDa (Hu et al. 2022b). Additionally, two enzymes from *P. multocida*, PmHS1 and PmHS2, also synthesize heparosan. PmHS1 mainly produces high-molecular-weight heparosan, while PmHS2 tends to synthesize low-molecular-weight ones (Otto et al. 2012).

In addition to the above strategies, heparosan lyase ElmA, which specifically cleaves the glycosidic bonds of heparosan, can also be used. By adopting a "synthesis followed by truncation" approach, heparosan with molecular weights ranging from 0.40 kDa to 1.90 kDa can be obtained (Xi et al. 2024; Barreteau et al. 2012).

In general, precursor pools such as UDP-GlcA and UDP-GlcNAc were most directly enhanced to improve yield; however, their positive effect on molecular weight was contingent on the concurrent optimization of the polymerization rate and the probability of termination (Nehru et al. 2021). A correlation between heparosan yield and molecular weight was observed. For example, in *B. subtilis* 168, endogenous upregulation of *tauD* resulted in a 55% increase in heparosan production and significantly affected the molecular weight and polydispersity of the resulting heparosan (Jin et al. 2016). By increasing the supplies of UDP-GlcA and UDP-GlcNAc within the optimal window of cell density and energy state, followed by the introduction of controllable polymerase expression, the target molecular weight and distribution uniformity were controlled without a significant effect on yield. In summary, a high precursor supply was combined with appropriate PmHS-type selection, and a phased dynamic strategy was adopted to sequentially optimize yield, chain length, and homogeneity at different fermentation stages, thereby achieving improved overall performance and scalability.

#### Compliance by design: safe hosts and antibiotic-free, GMP-ready constructs

As a key precursor to pharmaceutical heparin, the production of heparosan was required to minimize clinical and manufacturing risks at the source; past heparin contamination incidents highlighted the necessity of end-to-end safety, traceability, and quality control (Kishimoto et al. 2008). Under the U.S. Food and Drug Administration (FDA)’s Generally Recognized as Safe (GRAS) framework, microbial preparations were considered “generally recognized as safe” when qualified experts concluded, based on scientific procedures or a history of safe use, that its intended conditions of use presented a reasonable certainty of no harm, and dossiers were typically expected to cover taxonomy, genome characterization, absence of toxins and antimicrobial resistance, manufacturing controls, and intended exposure (Burdock and Carabin 2004). Host eligibility was assessed by whole-genome analysis to confirm the absence of toxins and mobile elements carrying resistance genes, and by functional assays to verify that virulence phenotypes had been eliminated (Pal et al. 2024). To mitigate the risk of horizontal gene transfer, antibiotic selection markers were avoided by employing markerless integrations or recyclable systems (*Cre*/*lox*P, FLP/FRT, I-*Sce*I) that yielded scar-free genomes, and were complemented with counter-selection strategies (sacB, rpsL) or CRISPR-assisted allelic exchange to enable efficient and precise edits (McLellan et al. 2017; Ma et al. 2024). Under Good Manufacturing Practice (GMP), master and working cell banks were required to have a traceable lineage, to undergo genomic identity testing, and to be tested for sterility and mycoplasma; acceptance criteria were established for adventitious agents and genetic drift, and validated analytical methods for endotoxin, host-cell proteins, and residual DNA were aligned with parenteral specifications (Gebo et al. 2021). Given the endotoxin risk inherent to Gram-negative hosts, upstream lipopolysaccharide attenuation, such as *lpxM* or *msbA* engineering, was implemented in conjunction with efficient downstream clearance to ensure that heparosan products met pharmaceutical limits (Wu et al. 2024; Zhou et al. 1998). In summary, to ensure that heparosan, a key precursor to heparin, was safe, controllable, and traceable, and met injectable specifications, a closed-loop system for end-to-end risk minimization and quality control was established under GRAS principles and GMP systems. This system encompassed host selection and marker-free gene construction, whole-genome and functional safety verification, cell bank establishment and quality release, and both source attenuation and downstream removal of impurities, including endotoxins.

## Chemoenzymatic production of heparin

### Modification enzymes in heparin biosynthesis

**Fig. 3:**
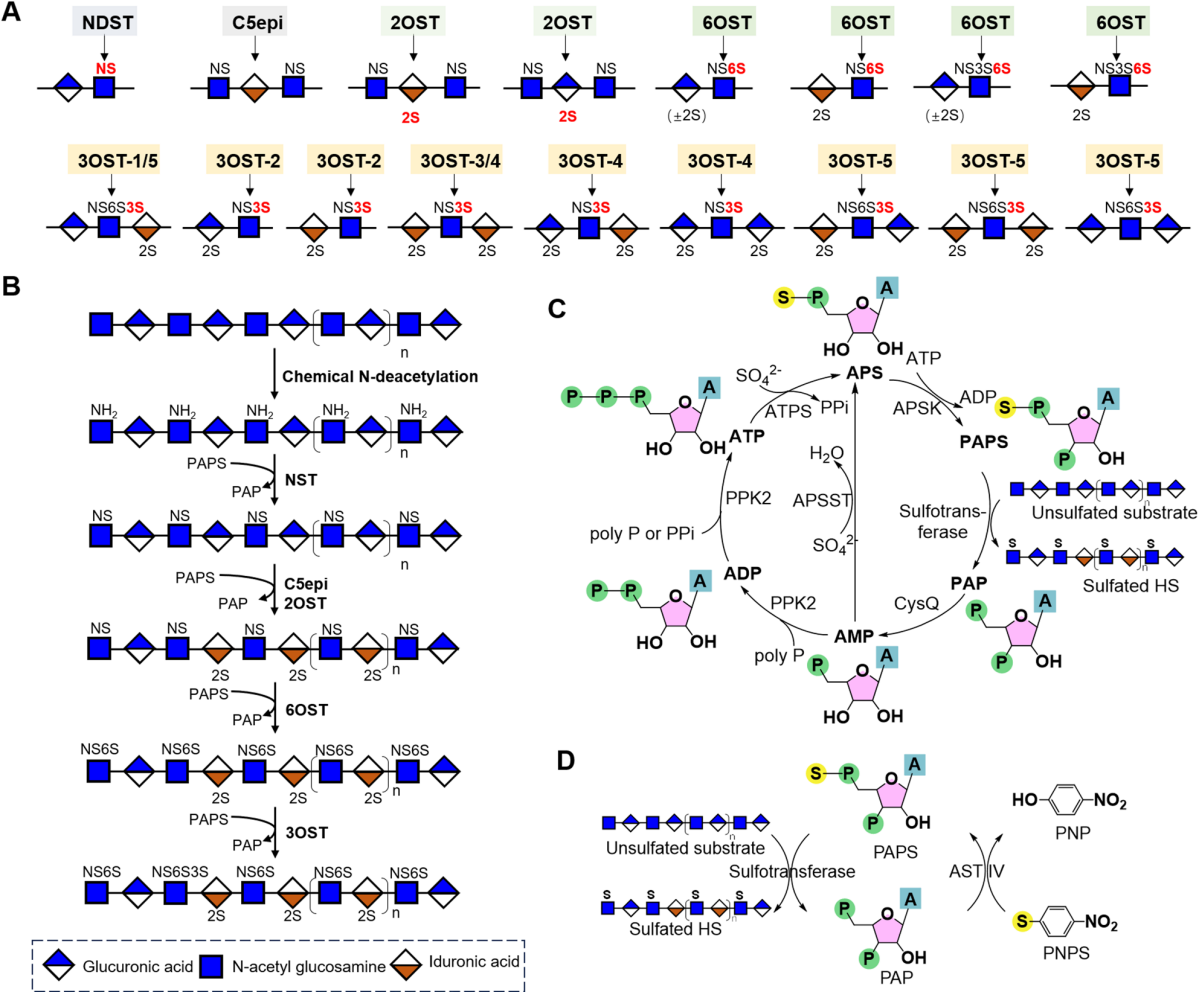
Schematic illustration of heparosan modification and in vitro PAPS regeneration. The modification sites of the sulfotransferases in heparin biosynthesis. **B** Schematic of heparin production from heparosan. Heparosan is chemically N-deacetylation and enzymatically modified into heparin by a series of enzymes, including NST, C5epi, 2OST, 6OST, and 3OST. **C**–**D** In vitro PAPS regeneration. The PAPS regeneration system provides the sulfate groups required for the sulfation reactions during heparin biosynthesis

Five kinds of heparin modifying enzymes, including *N*-deacetylsulfotransferase (NDST), C5-epimerase (C5epi), 2-*O*-sulfotransferase (2OST), 6-*O*-sulfotransferase (6OST) and 3-*O*-sulfotransferase (3OST) (Fig. 3A). Under the catalysis of these enzymes, the polysaccharide chains are modified to heparin structures, thereby playing multiple biofunctions.

#### NDST

NDST is a bifunctional enzyme composed of a deacetylation domain and an *N*-sulfation domain (Wei et al. 1993). It catalyzes two consecutive and coupled reactions, including (1) hydrolysis of the acetyl group from the C-2 position of GlcNAc, producing glucosamine (GlcNH₂) residues, and (2) transferring a sulfate group (-SO₃⁻) from 3'-phosphoadenosine 5'-phosphosulfate (PAPS) to the free amino group of GlcNH₂, finally producing *N*-sulfated glucosamine (GlcNS). This is a rate-limiting step in heparin biosynthesis as its product is the "essential substrate" for the subsequent reactions.

There are four NDST isoforms (NDST1-4) in mammals, and each has unique tissue distribution and substrate specificities that shape the structural heterogeneity of heparin. NDST1 and NDST2 are widely expressed, with NDST1 dominant in most tissues and NDST2 dominant in mast cells (Pikas et al. 2000). They are the primary isoforms for forming N-sulfated domains, which is critical for many physiological processes such as embryonic patterning, cell proliferation, blood coagulation, and tissue repair. Notably, overexpression of *NDST2* in HEK293 cells increased heparin chain lengths, suggesting that NDST2-specific substrate modification stimulates heparin elongation (Deligny et al. 2016). NDST3 is mainly expressed in brain, but also found in kidney, liver, and lung tissues (Aikawa and Esko 1999). NDST4 is restrictedly expressed in the adult brain and throughout embryonic development (Aikawa et al. 2001). NDST3 has relatively higher N-deacetylase activity and lower sulfotransferase activity. In contrast, NDST4 exhibits peculiar sulfotransferase activity and very low N-deacetylase activity (Li et al. 2018).

Structural and functional characterization of NDST1 has been achieved via crystal and cryo-electron microscopy analyses (Saribaş et al. 2004; Mycroft-West et al. 2024). This protein comprises a non-catalytic N-terminal domain, a deacetylase domain, and a sulfotransferase domain, forming an elbow-shaped structure. The deacetylase domain contains a typical deacetylase His-His-Asp triad, which is associated with metallic cations (Mycroft-West et al. 2024). The sulfotransferase domain consists of heparin substrate binding cleft near the PAPS and PAP binding sites (Kakuta et al. 1999). It consecutively sulfates the substrate from the nonreducing end to the reducing end, generating a cluster of *N*-sulfated glucosamine residues (Sheng et al. 2011). The deacetylase domain and sulfotransferase domain adopt a back-to-back topology, with their active sites oriented in opposite directions, hindering direct transfer of heparin intermediates from one domain to another.

To obtain active NDST1 for heparin biosynthesis, various expression strategies have been implemented, including the use of a baculovirus-insect cell and *Saccharomyces cerevisiae* expression systems (Dou et al. 2015). However, the heterologous expression of highly active recombinant NDSTs remains a widely recognized challenge. To address this challenge, a truncated protein containing the N-sulfotransferase (NST) domain has been expressed. To improve the activity of the recombinant NST, site-directed mutagenesis guided by multiple sequence alignment and structural analysis yielded a variant *N*-sulfotransferase (Mut02) with drastically enhanced half-life (105-fold increase) and catalytic activity (1.35-fold increase) (Xi et al. 2023). Furthermore, using Protein Repair One-Stop Service-Focused Rational Iterative Site-specific Mutagenesis (PROSS-FRISM), an engineered *N*-sulfotransferase with significantly improved stability (11.32-fold increase) and catalytic activity (2.53-fold increase) was achieved (Deng et al. 2024). Nevertheless, obtaining full-length NDSTs remains critical, particularly for reconstruction of the heparin biosynthetic pathway in a heterologous host. To achieve active recombinant NDST, the following potential strategies may be applicable: (1) Optimization of heterologous expression systems, such as the yeast and insect cell expression systems. (2) Screening of fusion tags (e.g., MBP and SUMO) to improve the solubility and expression level of the recombinant proteins. (3) Co-expression of chaperone proteins to facilitate proper protein folding. (4) Implementation of directed evolution strategies, especially those integrated with artificial intelligence-assisted design.

#### C5epi

C5epi modifies the heparin chain after the initial *N*-deacetylation/*N*-sulfation by NDST. It catalyzes the isomerization of GlcA residues adjacent to GlcNS into iduronic acid (IdoA) residues (Li et al. 1997). This reaction is reversible in vitro but irreversible in vivo (Hagner-McWhirter et al. 2004). The reversibility of the enzyme could be influenced by substrate structure. If GlcNS or GlcNH_2_ is present at the position that is three residues away from the modification site toward the nonreducing end or this position is unoccupied, C5epi catalyzes a reversible epimerization. On the other hand, if GlcNAc exists at the same position, the epimerization of GlcA to IdoA is irreversible. Further modification of IdoA at the epimerization site with 2-*O*-sulfations also prevents the reverse epimerization and leads to an accumulation of sulfated IdoA in heparin (Sheng et al. 2012). C5epi is expressed in multiple mammalian tissues, including the liver, lung, and spleen (Li 2010). The IdoA-containing domains generated by C5epi serve as binding sites for numerous proteins (e.g., growth factors and chemokines), thereby participating in the processes such as angiogenesis, embryonic development, and coagulation (Kreuger et al. 2006).

C5epi homologs derived from other species were also able to catalyze similar epimerization reactions, such as RED-C5epi, an epimerase from the marine bacterium *Bermanella marisrubri* (Raedts et al. 2013). Compared with mammalian C5epi, RED-C5epi shows less selectivity to substrates with or without N-sulfation but exhibits poor conversion efficiency. Another peculiar C5epi is derived from the snail *Achatina fulica*, which only epimerizes GlcA to IdoA in non-sulfated substrates like heparosan and desulfated N-acetylated heparin (Mochizuki et al. 2015).

Structural analysis revealed that C5epi primarily adopts a tight dimeric configuration, stabilized by interactions between the N-terminal β-hairpin and C-terminal α-helical barrel regions of each subunit (Debarnot et al. 2019). The negatively charged substrate binds within a positively charged cleft in each subunit. The catalytic mechanism of C5epi involves a two-step proton transfer process mediated by key active-site residues. First, a conserved tyrosine (Tyr578 in human C5epi) functions as a general base to abstract the proton from the C5 position of GlcA, forming a resonance-stabilized enolate intermediate. Then, the intermediate undergoes a conformational change, enabling a glutamate residue (Glu499 in human C5epi) to donate a proton to the opposite face of the C5 atom, ultimately producing the epimerized product of IdoA (Qin et al. 2015; Sheng et al. 2012).

#### 2OST

2OST transfers a sulfate group from PAPS to the 2-OH of uronic acid residues (IdoA or GlcA) flanked by GlcNS residues, with a preference for IdoA (Kobayashi et al. 1996). Only one isoform of 2OST is present in mammals. It is expressed in a wide range of human tissues, including the heart, brain, placenta, lung, liver, skeletal muscle, kidney, and pancreas (Kakuta et al. 1999; Kobayashi et al. 1999). The resulting 2-*O*-sulfation of 2OST contributes to the formation of specific sulfated domains in heparin chains, which are essential for interactions with a variety of proteins, including growth factors, chemokines, cytokines, and ECM proteins, to regulate numerous physiological processes (Kreuger et al. 2001; Witt et al. 2013; Nikolovska et al. 2015).

The crystal structure of 2OST reveals a homotrimeric architecture, with substrate-binding clefts located at the interface of adjacent monomers. The minimum substrate chain length required for 2OST to function has been determined to be a pentasaccharide. When binding to the enzyme, the substrate attaches with its reducing end at the top of 2OST and its non-reducing end at the N-terminal side, which is anchored to the membrane (Liu et al. 2014; Bethea et al. 2008). As the active site of 2OST selectively bind the uronic acid with ^4^C_1_ conformation, rather than the ^1^C_4_ or ^2^S_0_ conformations, allowing the sulfation on both IdoA and GlcA residues. Moreover, by mutating key amino acids involved in substrate recognition, 2OST variants with selective substrate preferences have been generated. For instance, the R189A variant exhibits a preference for GlcA-containing substrates, while the Y94A and H106A variants prefer IdoA-containing substrates. Notably, 2OST interacts with C5epi, and this interaction is essential for the formation of contiguous IdoA2S-containing disaccharides (Pinhal et al. 2001).

#### 6OST

6OST catalyzes the sulfation of glucosamine residues (GlcNS/GlcNAc) at the 6-position, with a preference for the GlcNS residues (Zhang et al. 2001). In mammals, three 6OST isoforms have been identified, namely 6OST-1, 6OST-2, and 6OST-3. Although each isoform displays distinct tissue-specific expression patterns (Habuchi et al. 2000; Jemth et al. 2003), advancing research has revealed no major differences in their substrate specificities (Xu et al. 2017). Owing to the high binding affinity of 6-*O*-sulfated heparin with a broad spectrum of proteins, such as growth factors (e.g., vascular endothelial growth factor, VEGF), chemokines (e.g., CXCL12), and enzymes (e.g., heparanase), the products of 6-OST are critical for a range of biological activities, including angiogenesis, cell migration, embryonic development, and anticoagulation (Schlessinger et al. 2000).

The structural and catalytic characteristics of 6OST have been elucidated through analysis of the crystal structure of zebrafish 6OST-3. This protein contains a canonical α/β sulfotransferase domain, featuring a strand-loop-helix motif (β1–α2) involved in PAPS binding, and a conserved α-helix (α4) that interacts with both PAP and the saccharide substrate. Accordingly, through site-directed mutagenesis, 6OST mutants (e.g., K132E, R206A, R329A, R116A) with enhanced sulfotransferase activity have been obtained (Xu et al. 2017).

#### 3OST

3OST transfers a sulfate group to the 3-OH of *N*-sulfo-D-glucosamine (GlcNS) or 6-*O*-sulfated N-sulfo-D-glucosamine (GlcNS6S), thereby forming 3-*O*-sulfated *N*-sulfo-D-glucosamine (GlcNS3S) or 3,6-di-*O*-sulfated *N*-sulfo-D-glucosamine (GlcNS6S3S) residues in heparin. Although 3-*O*-sulfated heparin accounts for approximately 0.5% of the total sulfation in the heparin chain, it is involved in a variety of biological functions, including anticoagulation, anti-inflammation, mediation of viral invasion, and modulation of neurite outgrowth (Karlsson et al. 2021; Huang et al. 2015; Tiwari et al. 2004; Thacker et al. 2014). There are seven different isoforms of 3OSTs encoded in the human genome, namely 3OST-1, -2, -3A, -3B, -4, -5, and -6. These 3OST isoforms exhibit different substrate specificities, which in turn display distinct biological functions (Liu and Pedersen 2022).

3OST-1 is the first discovered 3OST isoform in humans, which is predominantly expressed in the heart, brain, lung, and kidney (Shworak et al. 1999). Biochemical studies have shown that 3OST-1 specifically sulfates the GlcNS6S residue flanked by a GlcA unit on its nonreducing side and an IdoA2S unit on its reducing side (-GlcA-GlcNS6S-IdoA2S-) (Moon et al. 2012; Liu and Pedersen 2022). The 3-*O*-sulfated product of 3OST-1 exhibits high affinity for antithrombin Ⅲ (AT-Ⅲ), thus enabling heparin to exert anticoagulant activity (Kuberan et al. 2003b).

3OST-2 is predominantly expressed in brain, though low levels of expression have also been detected in the heart, placenta, lung, and skeletal muscle (Shworak et al. 1999). This enzyme catalyzes the sulfation of glucosamine residues that are linked to 2-*O*-sulfated uronic acid units at their nonreducing end, such as the motifs of GlcA2S-GlcNS and IdoA2S-GlcNS (Liu et al. 1999). The 3-*O*-sulfated products generated by 3OST-2 interact with the surface envelope glycoproteins of herpes simplex virus (HSV); this interaction directly mediates HSV entry into host cells (O’Donnell et al. 2006). Additionally, emerging evidence suggests that the product of 3OST-2 may act as a molecular chaperone that facilitates the abnormal phosphorylation of tau protein, a key pathological event closely associated with the development and progression of Alzheimer’s disease (Huynh et al. 2022).

3OST-3A and 3OST-3B share high sequence similarity in their sulfotransferase domains (Shworak et al. 1999). Consequently, they exhibit identical substrate specificity with preference of the glucosamine that is flanked by IdoA2S residues (IdoA2S-GlcNS-IdoA2S-) (Moon et al. 2012; Wander et al. 2021). Although 3OST-3 sulfotransferases are broadly expressed in many tissues, 3OST-3A has the highest expression in heart and placenta, whereas 3OST-3B is abundantly expressed in liver and placenta. The 3-*O*-sulfated products generated by 3OST-3 enzymes function as entry receptors for HSV, attributed to their high binding affinity for the viral glycoprotein D (Moon et al. 2004; Hubbard et al. 2010).Of note, an octasaccharide modified by 3OST-3 exhibited comparable binding affinity to antithrombin and similar IC₅₀ for inhibiting factor Xa activity, but with more rapid clearance kinetics than fondaparinux in a mouse model (Wang et al. 2017).

3OST-4 is exclusively expressed in the brain. It preferentially acts on glucosamine residues lacking 6-*O*-sulfation, which are flanked by 2-*O*-sulfated uronic acid units, like the motifs of -IdoA2S-GlcNS-IdoA2S-, -GlcA2S-GlcNS-IdoA2S- and -GlcA2S-GlcNS-GlcA2S- (Li et al. 2021). In addition to serving as the receptor of HSV (Hubbard et al. 2010; Ohka et al. 2021), the 3-*O*-sulfated products generated by 3OST-4 exhibit potent anticoagulant activity but weak binding affinity for platelet factor 4 (PF4) (Karlsson et al. 2021; Li et al. 2021). Furthermore, it shows high binding affinity for tau protein, thereby potentially regulating the pathological processes of Alzheimer’s disease (Li et al. 2021).

3OST-5 is primarily expressed in skeletal muscle and fetal brain (Xia et al. 2002). It exhibits broad substrate tolerance but shows a strong preference for the 6-*O*-sulfated glucosamine (GlcNS6S) residues flanked by GlcA residues in the sequence of -GlcA-GlcNS6S-GlcA-, a moderate preference for the GlcNS6S linked to GlcA on one side and IdoA2S on the other (-GlcA-GlcNS6S-IdoA2S- and -IdoA2S-GlcNS6S-GlcA-). It also acts on the GlcNS6S residues flanked by IdoA2S on both sides (-IdoA2S-GlcNS6S-IdoA2S-) (Wander et al. 2021; Chen et al. 2003). The product of 3OST-5 can not only exert anticoagulant activity but also mediate the entry of HSV (Xia et al. 2002). 3OST-6 is predominantly expressed in the kidney and liver, and its function remains poorly studied (Xu et al. 2005).

Crystallographic studies on 3OST-1, 3OST-3, and 3OST-5 have revealed high structural similarity among these three isoforms. They all adopt a roughly spherical conformation and contain a large open cleft, which is predominantly composed of positively charged amino acid residues and responsible for substrate binding (Xu et al. 2005; Moon et al. 2004). However, these three enzymes exhibit distinct substrate binding modes that determine their unique substrate specificities. For instance, in 3OST-1, the IdoA2S residue at the reducing side of the receptor unit adopts a ^1^C_4_ chair conformation, whereas the IdoA2S bound to 3OST-3 adopts a ^2^S_0_ skew-boat conformation. Additionally, the presence of a 2-*O*-sulfo group on the residue at the nonreducing side of the target unit is critical for binding to 3OST-3, whereas this 2-*O*-sulfo group is absent in the corresponding position for 3OST-1. In 3OST-1, two residues, Glu88 and His271, form a “gate” structure that narrows the substrate-binding cleft to a width of 6.7 Å. In contrast, the corresponding residues in 3OST-3 and 3OST-5 have much smaller side chains, resulting in a wider cleft (approximately 14.2 Å). Mutations of these amino acids in 3OST-1 (H271G and E88G/H271G) not only increased enzymatic reactivity (17-fold and 9-fold, respectively), but also altered the substrate specificity (Xu et al. 2008). Notably, the structure of α-helix 6 also influences substrate specificity among 3OST isoforms. The α-helix of 3OST-1 (spanning Pro153–Lys171) is longer than that of 3OST-3 (Pro245–Lys259), enabling additional interactions with the reducing end of the substrate (Moon et al. 2012).

### Chemoenzymatic modification of heparosan

Heparosan, the bacterial capsular polysaccharide, has been employed as the precursor for bioengineered heparin. Due to the challenge of achieving adequate expression levels and catalytic activity of recombinant NDST, chemical deacetylation has been incorporated into the process of producing heparin from heparosan (Zhang et al. 2008). Since the subsequent steps following chemical deacetylation are typically catalyzed by enzymes, the entire process is referred to as chemoenzymatic synthesis (Fig. 3B).

The chemoenzymatic modification of heparosan begins with the chemical *N*-deacetylation, which uses an aqueous solution of sodium hydroxide to remove the acetyl groups attached to the amino group of glucosamine residues (Saribaş et al. 2004; Dou et al. 2015; Kuberan et al. 2003b). Of note, excessively strong alkaline conditions can cause depolymerization of the starting material, resulting in shorter polysaccharide chains (Wang et al. 2011a). To avoid the depolymerization of polysaccharides, a relatively mild *N*-deacetylation method has been developed by incorporating *N*-trifluoroacetylhexosamine (GlcNTFA), instead of GlcNAc, into the synthesis of engineered heparosan. A LiOH solution (pH 12) was used for chemical deacetylation. Then, pH was adjusted to 7.0 for the subsequent *N*-sulfation, which could be accomplished through either chemical or enzymatic approaches. Chemical *N*-sulfation is performed using trimethylamine sulfur trioxide to generate *N*-sulfated heparosan polysaccharide (Kuberan et al. 2003a; Wang et al. 2011b). By controlling the chemical reaction time, bioengineered heparin with an *N*-acetyl/*N*-sulfo ratio similar to animal-sourced pharmaceutical heparin (12.7% *N*-acetylglucosamine and 82.9% *N*-sulfoglucosamine) has been obtained. Enzymatic *N*-sulfation is catalyzed by recombinant NST, which transfers the sulfo group from PAPS to the amino group of GlcNH₂ residues (Deng et al. 2024).

The next step is epimerization, catalyzed by C5epi. Recombinant C5epi converts GlcA in *N*-sulfated heparosan polysaccharides to IdoA through stereochemical rearrangement of the C5 hydroxyl group (Kuberan et al. 2003a). As this reaction is reversible, concomitant 2-*O*-sulfation is usually employed to enhance the efficiency (Chen et al. 2005). Notably, IdoA residues accounts for 70–80% of total uronic acids in heparin. Therefore, controlling the epimerization ratio is critical for producing bioengineered heparin that closely resembles porcine intestinal heparin.

Following epimerization and 2-*O*-sulfation, the final two steps in the chemoenzymatic synthesis of heparin involve 6-sulfation and 3-*O*-sulfation, depending on which 3OST isoform is used. In most cases, 6-*O*-sulfation precedes 3-*O*-sulfation, with 3OST-1 subsequently used to modify the 6-*O*-sulfated heparosan, thereby yielding heparin with anticoagulant activity (Deng et al. 2024). In addition, heparin polysaccharides with FGF2 binding ability or HSV binding ability have also been synthesized via adjusting the sequence of multi-enzyme cascade reactions (Chen et al. 2005).

### Chemoenzymatic synthesis of structurally defined heparin

Synthetic oligosaccharides with defined chain lengths have also been used as precursors for biosynthesis of structurally homogeneous heparin, a valuable asset for pharmaceutical applications. This process begins with the elongation of starting units, followed by a series of enzymatic modifications. Initially, a disaccharide (GlcA-AnMan) has been used as the starting unit for heparin oligosaccharide synthesis. Elongation of this disaccharide yields the heparosan backbones. However, monitoring and purifying the sugar chain during the synthetic processes are highly challenging and require considerable expertise. Subsequently, 1-*O*-p-nitrophenyl (pNP) glucuronide was introduced as an alternative starting unit. The pNP moiety exhibits specific UV absorbance at 310 nm, a property that significantly simplifies the detection and purification of both intermediates and final products. Notably, the pNP group can be removed after synthesis, producing heparins without any extraneous tags (Xu et al. 2014; Zhang et al. 2017c). In addition, azido- or tert-butyloxycarbonyl (Boc)-tagged units have also been successfully employed as starting units for chemoenzymatic synthesis of heparin, enabling construction of glycoconjugates or microarrays, or simplifying purification processes (Cai et al. 2014; Wu et al. 2013; Zhang et al. 2017b).

Elongation of the starting units is carried out by bacterial glycotransferases, such as *N*-acetylglucosaminyl transferase KfiA and KfiC from *E. coli* K5, or PmHS2 from *P. multocida*, yielding heparin backbones with defined length (Zhang et al. 2017b; Deng et al. 2022). To address the limitations of NDST, GlcNTFA or *N*-difluoroacetylglucosamine (GlcNDFA), which are easily converted to GlcNH₂ under mild alkaline conditions, have been used as substrates to replace the GlcNAc residues to incorporate into the elongated chains (Xu et al. 2011; Cao et al. 2024). After chemical *N*-deacetylation, the oligosaccharide is subjected to *N*-sulfation, which is catalyzed by the recombinant NST. The resulting *N*-sulfated oligosaccharide then undergoes enzymatic epimerization, 2-*O*-sulfation, 6-*O*-sulfation, and 3-*O*-sulfation, ultimately producing structurally defined heparins (Xu et al. 2011). Using this strategy, numerous structurally defined ULMW heparins with potent anticoagulant activity have been generated (Moon et al. 2012; Xu et al. 2014, 2011, 2012; Whelihan et al. 2016; Arnold et al. 2020). Notably, the synthesized 6-mer heparin closely resembles fondaparinux, while the 12-mer can be neutralized by protamine, a U.S. FDA-approved drug for heparin reversal (Xu et al. 2014).

### Optimization of chemoenzymatic reaction systems

To further enhance synthetic efficiency, scalability, and cost-effectiveness, efforts have been made to optimize chemoenzymatic reaction systems, including the incorporation of PAPS regeneration systems, the development of one-pot synthesis systems, and the immobilization of biosynthetic enzymes.

#### In vitro PAPS regeneration system.

Heparin, an important anticoagulant, undergoes a critical synthesis process involving the sulfation of glycosaminoglycan chains, which is strictly dependent on PAPS as the exclusive active sulfonate donor (Schwartz and Domowicz 2014; Xu et al. 2023; Zhao et al. 2025). The chemical structure of PAPS consists of adenosine, two phosphate groups, and a sulfate group. In vivo, PAPS biosynthesis occurs predominantly via the cysteine metabolic pathway (Xu et al. 2024; Koprivova and Kopriva 2016). However, PAPS faces intrinsic limitations such as low cellular accumulation and poor stability in vitro, making it difficult to meet the needs of industrial production. Therefore, developing efficient in vitro regeneration systems has become a pivotal research focus to overcome this bottleneck (Fig. 3C). Currently, the in vitro regeneration methods for PAPS are primarily divided into “PAP-dependent” enzymatic regeneration methods and “ATP-dependent” bifunctional enzyme cascade reaction systems (Gu et al. 2025; Liu et al. 2023a, 2021).

“PAP-dependent” enzymatic regeneration is the most commonly used method for in vitro regeneration of PAPS utilizing the catalytic activity of sulfotransferases (Fig. 3D). This method uses PAP, a byproduct of the sulfation reaction, as the substrate, transferring the sulfate group from pNP sulfate (pNPS) to PAP by sulfotransferase, thereby generating PAPS and enabling the recycling of byproducts (Datta et al. 2020; Malojcić et al. 2008). This method effectively addresses the inhibitory effect of PAP on sulfotransferases in traditional systems. Discovered sulfotransferases, such as human ASTIV (such as Hsult1A1/1A2/1A3) and Mus SULT1D1 have the similar structural characteristics. The flexible gating loop at the entrance of their substrate-binding region acts like a “cap,” easily blocking the entry of large, negatively charged PAP molecules, thereby impairing catalytic efficiency(Berger et al. 2011; Gamage et al. 2003; Dombrovski et al. 2006). However, the mechanism of the gating loop remains incompletely understood. Furthermore, the internal disulfide bonds of ASTIV often misfold in *E. coli* expression system, resulting in low soluble expression levels. Enzyme activity and expression have been significantly improved through signal peptides to direct enzyme secretion into the periplasm (Zhou et al. 2019).

“ATP-dependent” bifunctional enzyme cascade reaction system is the most mature technology (Fig. 3B), which relies on the synergistic action of ATP sulfurylase and APS kinase (Venkatachalam 2003). In the first step, ATP sulfurylase catalyzes the binding of ATP to sulfate to form 5'-phosphoadenosine sulfate (APS). In the second step, catalyzed by APS kinase, APS binds to another molecule of ATP to form PAPS. Through the cascade action of these two enzymes, ATP is directly converted to PAPS. However, this reaction is susceptible to feedback inhibition by the intermediate product ADP, and the high cost of ATP renders it unsuitable for industrialization (Mueller and Shafqat 2013). To overcome this problem, researchers have innovatively established a closed-loop ATP regeneration system based on polyphosphate kinase (PPK) (Xu et al. 2021). This system, through the introduction of PPK and the use of pyrophosphate (PPi) as a phosphate donor, efficiently phosphorylates the byproduct ADP into ATP, which is then converted to PAPS through a bifunctional enzyme, forming a cyclic pathway of "ATP consumption-PAPS production-ATP regeneration". This design not only enables the recycling of ADP but also significantly reduces raw material consumption through a closed-loop reaction, providing a green and sustainable solution for the industrial production of PAPS. Recently, Gu et al. construct an RPA synthesis pathway, using adenine and D-ribose as raw materials to break through ATP-dependence of the traditional PAPS synthesis. With AMP (adenosine monophosphate) as the core intermediate carrier, the closed-loop process of “synthesizing AMP from cheap raw materials-converting AMP into PAPS-regenerating AMP after PAPS consumption” reduces ATP consumption and intermediate loss. At the same time, it is compatible with microbial intracellular expression systems and can directly couple the synthesis of downstream sulfated products (such as chondroitin sulfate A) (Gu et al. 2025).

#### One-pot synthesis of heparin

Unlike labor-intensive multienzyme cascade reactions, which could cause significant product loss due to multiple purification steps, the one-pot synthesis system integrates multiple enzymatic reactions into a single reaction vessel, thereby enhancing efficiency, reducing costs, and facilitating scalability. Although the total one-pot synthesis of heparin has not yet been achieved, methods that incorporate multiple steps into a single reaction vessel have been established. For instance, by integration of in situ UDP-GlcNTFA generation with heparin backbone elongation in one-pot, Wu et al. synthesized a biotinylated heparosan hexasaccharide (Wu et al. 2015). By optimizing processing conditions and ratio of starting unit and glycosyl donors (UDP-GlcNTFA and UDP-GlcA), Chandarajoti et al. obtained a narrow-size distribution oligosaccharides through a one-pot reaction (Chandarajoti et al. 2014).

Of note, a successful one-pot chemoenzymatic synthesis of sulfated polysaccharides has been achieved using *N*-sulfated heparosan as the substrate. Through modulating the enzyme to substrate (E:S) ratio, bioengineered heparin products with significant anticoagulant activity have been produced (Bhaskar et al. 2015). Despite the fact that the one-pot approach enables the final product to be acquired without intermediate isolation, thereby enhancing efficiency, the one-pot synthesis of heparin still faces substantial challenges, which stem from the sequential nature of its biosynthetic pathway, potential substrate inhibition, side reactions, and substrate competition arising from the coexistence of multiple enzymes. Incorporating strategies from heparin chemical synthesis (Kulkarni et al. 2018; Dey et al. 2020; Jeanneret et al. 2019), such as utilizing chemically synthesized building blocks during glycosyl chain elongation followed by enzymatic modification after the removal of chemical protecting groups, may provide an effective solution to the sequential synthesis challenge (Lu et al. 2018). Additionally, staged enzyme addition and microcapsule isolation are promising approaches worth exploring. Therefore, future research could integrate chemical and biocatalytic processes to develop a chemoenzymatic one-pot system, which would balance rapid construction with highly functionalized modifications to produce homogeneous heparin.

#### Immobilization of biosynthetic enzymes

Another strategy to improve the chemoenzymatic synthesis of heparin is the immobilization of biosynthetic enzymes, which addresses the inherent limitations of free enzyme systems, such as poor reusability, instability under reaction conditions, and high costs associated with downstream purification of enzymes from products. Immobilization of these biosynthetic enzymes typically utilizes solid supports. Agarose aldehyde beads and CNBr-activated Sepharose, for instance, have been shown to yield high immobilized activity for heparin-modifying enzymes and AST-Ⅳ immobilization (Xiong et al. 2013). Using immobilized C5epi and 2OST involved in heparin modification, together with AST-Ⅳ for cofactor recycling, *N*-sulfated, 2-*O*-sulfated heparosan has been successfully synthesized from *N-*sulfated heparosan (Xiong et al. 2013). Additionally, bioengineered heparin polysaccharides with binding affinity for antithrombin, fibroblast growth factor-2, and HSV envelope glycoprotein D have been generated by immobilizing sulfotransferases in conjunction with a PAPS regeneration system (Chen et al. 2005). Furthermore, gram-scale production of bioengineered heparin with similar disaccharide and tetrasaccharide compositions and comparable anti-Ⅱa and anti-Ⅹa activities to that of USP heparin has been achieved via immobilization of recombinant heparosan sulfotransferases, demonstrating the significant advantages of immobilized enzyme systems in the chemoenzymatic synthesis of animal-free heparin (Douaisi et al. 2024).

## De novo biosynthesis of bioengineered heparin

**Fig. 4:**
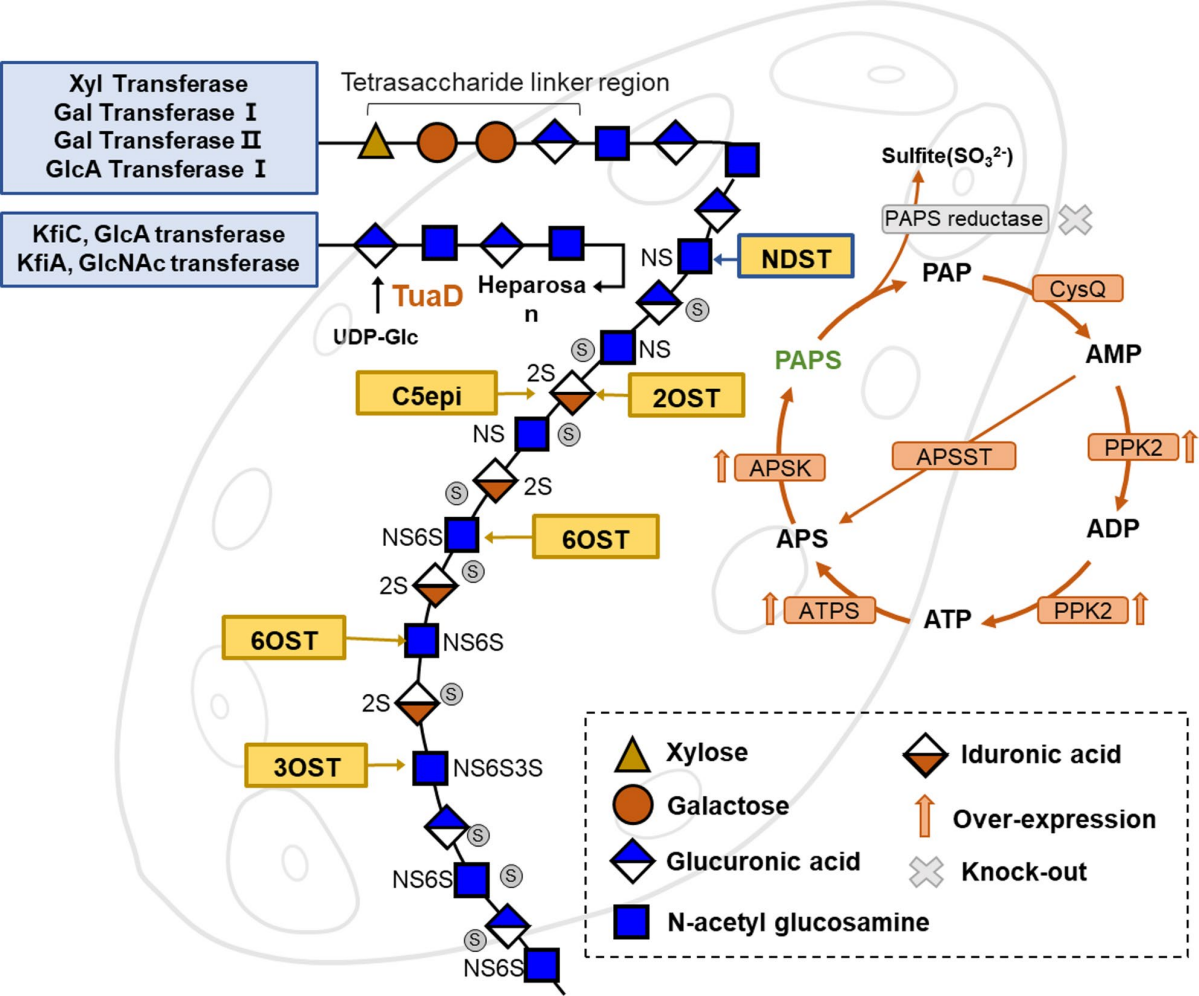
Schematic diagram of de novo heparin biosynthesis and in vivo PAPS supply

Commercial production of clinically essential sulfonated natural products, such as the glycosaminoglycans (GAGs) heparin and chondroitin sulfate, has historically been dependent on extraction from animal tissues. This paradigm presents significant challenges, including the potential for zoonotic pathogen contamination, supply chain instability arising from animal diseases, inherent batch-to-batch structural heterogeneity, and ethical concerns (Jiang et al. 2025; Sheng et al. 2024). In response, de novo biosynthesis via metabolically engineered cell factories has emerged as a compelling alternative, offering enhanced safety, product homogeneity, and scalability (Fig. 4).

### Mammalian biosynthesis

The production of bioengineered heparin using mammalian cell culture has seen significant advances through both metabolic engineering and bioprocess optimization (Table 2). Heparin is produced exclusively in granulated cells, such as mast cells, which are not suitable for commercial applications. While mastocytoma (MST) cells were readily grown in culture and synthesized sulfated heparosan, they lacked anticoagulant activity. Subsequent transfection of MST cells with a retroviral vector containing *3OST-1* restored anticoagulant activity (Gasimli et al. 2013). A multiplex genome engineering strategy was deployed to create MST cell lines that produced highly sulfated heparosan with anticoagulant activity exceeding pharmaceutical heparin. Using a gene expression profiling guided strategy, the biosynthetic pathways were engineered to eliminate production of contaminating chondroitin sulfate and produce highly anticoagulant heparin with a composition approximating porcine derived heparin (Thacker et al. 2022). The cell line was also successfully adapted to serum-free medium in anticipation of commercial production.

**Table 2:** Host cells, enzymes, and production levels for de novo biosynthesis of heparin

Host	Synthetic biology strategies	Production level	Sulfation degree	Activity	Molecular weight (kDa)	Reference
CHO-S	Exogenously expressing *NDST2* and *3OST-1*	~ 3.46 μg/mL	1S: 97.5% 2S: minor amount	/	/	Baik et al. 2012a, b
CHO-S	Developing Golgi-targeted *3OST-1* and enhancing *3OST-1* expression	~ 0.368 μg/mL	1S: 21.3% 2S: 7.4% 3S: 9.7%	∼137 U/mg	/	Datta et al. 2013
MST-10H	Increasing the expression of *3OST-1*	~ 0.18 μg/mL	1S: 10.0% 2S: 24.9% 3S: 63.3%	50–60 U/mg	10–12	Gasimli et al. 2013
CHO-S	Supplemental feeding to improve production yield and sulfation	∼90 μg/mL	1S: 92% 2S: 6%	/	/	Baik et al. 2015
HEK-293	Overexpressing serglycin in human cells	8.4–25.1 μg/mL	1S: 32% 2S: 34% 3S: 19%	/	/	Lord et al. 2016
MST cells	Overexpressing genes (*3OST-1*, *6OST-1*, *6OST-2*, and *NDST2*) and knocking out genes (*Csgalnact1*, *Csgalnact2*, and *Chsy1*)	6.0 μg/mL	1S: 9% 2S: 22% 3S: 65%	259–304 U/mg	25.7–33.0	Thacker et al. 2022
*P. pastoris*	Overexpressing heparin biosynthesis enzymes through Tag-fusion and N-terminal engineering (NDST, C5epi, 2OST, 6OST, and 3OST)	2080 μg/mL	1S: 0.77% 2S: 1.61% 3S: 1.99%	/	349	Liu et al. 2022

Although heparin is exclusively biosynthesized in granulated cells, such as mast cells, sulfated heparosan can be produced by nearly all animal cells (Karlsson et al. 2021). As part of ongoing efforts to bioengineer Chinese hamster ovary (CHO) cells for heparin production, co-introduction of *3OST-1* and *NDST2* resulted in the synthesis of sulfated heparosan with low anticoagulant activity (Baik et al. 2012a). Further genetic modification of CHO cells to overexpress key enzymes involved in the heparin biosynthetic pathway led to an approximately 100-fold increase in anticoagulant activity (Baik et al. 2015). To enhance the functional sulfation of heparosan, 3OST-1 was specifically engineered to target the Golgi apparatus, whose correct localization facilitated the formation of a single, high-affinity AT-binding site, resulting in high anti-factor Xa activity (137 ± 36 units/mg). Moreover, stable overexpression of *3OST-1* was associated with a significant increase in the levels of 2-*O*-, 6-*O*-, and *N*-sulfo group-containing disaccharides (Datta et al. 2013).

The presence of proteoglycans provides a natural scaffold that facilitates heparin biosynthesis (Fig. 4). In a recent study, HEK293 cells were transfected to express the heparin core protein serglycin, resulting in the production of sulfated heparosan with anticoagulant activity approximately one-seventh that of pharmaceutical heparin (Lord et al. 2016). Although this represents a significant step toward the recombinant production of bioengineered heparin, the structural characteristics of the produced sulfated heparosan still differ from those of pharmaceutical-grade heparin. These discrepancies highlight the need for further metabolic engineering to more closely mimic the highly sulfated and structurally defined features of native heparin.

### Microbial biosynthesis

De novo biosynthesis of heparin in microbial cells has recently been achieved (Table 2). Zhang et al. employed metabolic engineering to integrate genes responsible for heparin precursor synthesis and sulfation modules into the genome of *Pichia pastoris (Komagataella phaffii)*, successfully reconstructing the heparin biosynthesis pathway. Key enzymes, including the bifunctional NDST, C5epi, and various sulfotransferases (2OST, 6OST, and 3OST), wereeffectively expressed with high activity in *P. pastoris*. To enhance NDST enzyme activity, they performed an N-terminal truncation of 83 amino acids and fused multiple solubility-promoting tags, such as the maltose binding protein (MBP) tag, which significantly improved its expression and activity. Using a fed-batch fermentation strategy with methanol and other single-carbon sources, the engineered yeast cell factory efficiently expressed the relevant enzymes and achieved de novo biosynthesis of heparin. Optimizations led to a production level of 2.08 g/L of bioengineered heparin in a 3-L fermenter, demonstrating the potential for large-scale production (Gao et al. 2022). *P. pastoris* is a well-established eukaryotic expression system characterized by its robust protein expression capabilities, eukaryotic post-translational modification machinery, and suitability for high-density fermentation. It has been classified by the FDA as a GRAS host organism and is extensively utilized in the production of recombinant proteins and high-value biochemicals. Importantly, *P. pastoris* possesses an endogenous biosynthetic pathway for PAPS. This intrinsic capability makes it a promising host for the production of sulfated complex carbohydrates.

### Engineering of intracellular PAPS pathway

The de novo synthesis of heparin in microbial cell factories represents a promising strategy to replace conventional animal-derived extraction, offering significant potential for industrial-scale production. Synthetic pathways for the heparin precursor, heparosan, have been successfully established in various hosts, including *E. coli*, *B. subtilis*, and *P. pastoris* (Zhang et al. 2012, 2017a, 2022; Williams et al. 2019; Nehru et al. 2020; Chen et al. 2017). A principal metabolic bottleneck for the de novo biosynthesis of fully sulfated heparin in these microbial systems is the limited endogenous supply of the universal sulfonate donor, PAPS. Most microbial hosts (e.g., *E. coli* and *P. pastoris*) cannot naturally produce PAPS to high levels, a substance essential for the extensive polysulfation of heparin precursors (Bhaskar et al. 2012). Consequently, metabolic engineering strategies aimed at augmenting the intracellular PAPS pool are critical for achieving high-yield heparin production (Zhang et al. 2022).

A primary strategy for enhancing the PAPS biosynthetic pathway involves the overexpression of its key enzymes: ATP sulfurylase (ATPS), which forms adenosine-5'-phosphosulfate (APS) from ATP and inorganic sulfate, and APS kinase (APSK), which phosphorylates APS to yield PAPS (An et al. 2017; Li et al. 2025). Co-expression of these enzymes, often sourced from microorganisms known for high PAPS levels, has been shown to significantly increase intracellular PAPS concentrations (Datta et al. 2020). The successful implementation of this strategy in *P. pastoris* has enabled the production of various highly sulfated products, including chondroitin sulfate (Xiong et al. 2025; Jin et al. 2021), heparin (Liu et al. 2022), and sulfated cholesterol (Xiao et al. 2024), validating its efficacy as a cell factory for sulfated natural products.

Further enhancements to the PAPS supply involve advanced pathway engineering, such as modular pathway reconstruction and the optimization of process enzymes (Gu et al. 2025; Liu et al. 2023a; Xu et al. 2023). For example, Gu et al. explored novel sulfotransferases while designing and reconstructing a pathway for high-efficiency PAPS production from adenine and ribose (Gu et al. 2025). Similarly, Xiong et al. systematically engineered a multi-component PAPS regeneration system in *P. pastoris*, integrating modules for PAPS synthesis, ATP regeneration, and AMP recycling to successfully elevate the final chondroitin sulfate A sulfation degree to a remarkable 96% (Xiong et al. 2025). Complementary approaches include eliminating non-productive metabolic flux by knocking out or downregulating genes encoding PAPS-degrading enzymes (Badri et al. 2019; Thomas and Surdin-Kerjan 1997) and fine-tuning enzyme expression levels via promoter engineering and codon optimization to balance metabolic burden (Xiong et al. 2025). These comprehensive strategies have been successfully implemented in yeast platforms, progressively improving the sustainability and cost-effectiveness of the sulfation process. Although not yet employed, they are expected to further enhance the sulfation degree of the de novo biosynthesized heparin.

## Discussion

Bioengineered heparin represents a promising alternative to traditional animal-derived heparin, addressing many of the safety, supply, and ethical concerns associated with animal sources. Synthesized through microbial fermentation combined with chemoenzymatic modification, bioengineered heparin offers improved consistency, homogeneity, and scalability. Recent advances have been made in metabolic engineering strategies, notably integrating heparosan biosynthesis with precise enzymatic sulfation, allowing fine control over molecular weight and sulfation patterns. Enzyme engineering and immobilization techniques have enhanced catalytic activity and process efficiency, enabling multi-gram scale production of bioengineered heparin with biological and structural properties closely matching those of USP-standard porcine heparin.

Scaling bioengineered heparin production from laboratory to industrial volumes remains challenging across all emerging strategies. Hybrid approaches based on heparosan fermentation followed by chemoenzymatic maturation are currently the most advanced but are limited by downstream complexity: multi-step sulfation and epimerization reactions require expensive, difficult-to-produce enzymes and tight control of reaction conditions that are hard to maintain at large scale. These sequential modifications lead to long processing times, high buffer and reagent consumption, and substantial operational costs. Although upstream fermentation is readily scalable, the need to reproduce intricate sulfation patterns at industrial volumes imposes significant constraints on process robustness, cost reduction, and batch consistency. Fully de novo biosynthetic routes, which aim to reconstruct the entire heparin pathway in engineered microbes or mammalian cells, face even more fundamental obstacles. The metabolic burden of producing highly sulfated glycosaminoglycans, the difficulty of replicating ER/Golgi-like microenvironments, and the low titers observed to date severely limit industrial feasibility. Moreover, purification of highly anionic, viscous polysaccharides adds considerable cost at scale, and regulatory expectations for structural consistency and safety remain stringent. Together, these issues keep bioengineered heparin at an economic disadvantage compared with low-cost, animal-derived products. Overcoming these barriers will require major advances in enzyme engineering, continuous bioprocessing, metabolic pathway design, and analytical standards to make engineered heparin competitive in large-scale manufacturing. A systematic, critical comparison of their relative strengths, weaknesses, and state of maturity of these production methods is provided in Table 3.

**Table 3:** Comparison of bioengineered heparin production strategies

Criterion	Animal extraction	Heparosan → Chemoenzymatic Modification	De Novo Biosynthesis
Maturity	★★★★★★★ (Very high; fully industrialized)	★★★☆☆☆ (Medium; semi-industrial, still scaling)	★★☆☆☆☆ (Early-stage research)
Safety/Purity	Moderate (dependent on supply chain and raw materials)	High (more controllable upstream and downstream processes)	Highest in principle (fully defined biological system)
Structural Control	Low	Medium–High	Highest (theoretically programmable)
Cost Potential	Low (established production, low raw-material cost)	Medium (cost driven by enzyme production and modification steps)	Currently high (low titers, complex pathway engineering)
Sustainability	Low (animal dependent)	Moderate (fermentation-based precursor)	High (no animal input; renewable carbon sources)
Scalability	Fully achieved	Upstream scalable, downstream remains a bottleneck	Not yet scalable (low yields)
Regulatory Alignment	Fully established	Requires demonstration of equivalence and consistency	Very early (regulatory framework not yet applicable)

Bioengineered heparin faces critical challenges on its path to clinical translation, particularly regarding ensuring batch-to-batch structural consistency. Given the inherent structural complexity of heparin, maintaining precise control over critical quality attributes—including disaccharide composition, sulfation patterns, molecular weight distribution, and specific functional pentasaccharide sequences—is essential for reproducible biological activity. Advanced analytical techniques such as multidimensional NMR and LC–MS disaccharide mapping have become indispensable for characterizing these attributes and must be integrated into rigorous quality control frameworks to achieve pharmaceutical-grade consistency comparable to animal-derived heparin. Moreover, upstream metabolic engineering strategies to finely tune the expression and localization of biosynthetic enzymes, combined with real-time process monitoring and robust downstream purification, are required to minimize batch variability and ensure the safety and efficacy of bioengineered heparin batches (Baik et al. 2012b; Douaisi et al. 2024).

Another major translational barrier lies in mitigating immunogenicity risks attributed to non-human glycosylation and process-related impurities. Although bioengineered heparin production in humanized or microbial hosts theoretically eliminates concerns of animal-derived contaminants and pathogen transmission, it introduces potential new immunogenic epitopes linked to host-specific glycosylation patterns or residual host proteins. Comprehensive immunogenicity assessment—spanning in vitro assays for complement activation, platelet factor 4 binding, and leukocyte response, as well as in vivo evaluation in relevant animal models—is critical for safety de-risking. While preliminary data suggest that bioengineered heparin could reduce certain adverse immune reactions compared to traditional products, large-scale clinical validation is still pending. Regulatory agencies like the FDA and EMA emphasize thorough physicochemical and bioequivalence characterization alongside immunological safety profiling, signaling the need for integrated regulatory science approaches to bridge laboratory development with pharmacopoeial standards and facilitate clinical adoption of this promising therapeutic alternative.

In summary, bioengineered heparin production technologies have reached a stage poised for industrial application, offering a safer and more sustainable source of critical anticoagulant drugs, with future improvements expected to broaden therapeutic applications and improve production economics.

## Data Availability

Not applicable.

## Abbreviations

GAG: Glycosaminoglycan
UFH: Unfractionated heparin
LMWH: Low molecular weight heparin
ULMWH: Ultra-low molecular weight heparin
GlcA: Glucuronic acid
GlcNAc: *N*-Acetylglucosamine
UDP-GlcNAc: UDP-*N*-acetylglucosamine
UDP-GlcA: UDP-glucuronic acid
GalU: UDP-glucose pyrophosphorylase
GlmM: Phosphoglucosamine mutase
GlmU: A bifunctional enzyme that acetylates glucosamine-1-phosphate to *N*-acetylglucosamine-1-phosphate and then catalyzes the uridyltransferase (pyrophosphorylase) reaction to yield UDP-GlcNAc
Pgm: Phosphoglucomutase
UDP-Glc: UDP-glucose
KfiD: UDP-glucose dehydrogenase
KfiA: An α1 → 4 *N-*acetylglucosaminyltransferase
KfiC: A β1 → 4 glucuronyltransferase
PmHS: A single bifunctional heparosan synthase
KpsM: Inner-membrane permease
KpsT: ATP-binding subunit
Kdo: 3-Deoxy-D-manno-oct-2-ulosonic acid
KpsC and KpsS: Kdo transferases
KpsM: The multispanning inner membrane channel
KpsT: The cytosolic ATP-binding subunit
ECM: Extracellular matrix
NDST: *N-*Deacetylsulfotransferase
C5epi: C5-epimerase
2OST: 2-*O*-Sulfotransferase
6OST: 6-*O*-Sulfotransferase
3OST: 3-*O*-Sulfotransferase
GlcNH₂: Glucosamine
PAPS: 3'-Phosphoadenosine 5'-phosphosulfate
APS: 5'-Phosphoadenosine sulfate
GlcNS: *N*-Sulfated glucosamine
GlcNTFA: *N*-Trifluoroacetylhexosamine
CHO: Chinese hamster ovary
HSV: Herpes simplex virus
pNP: P-nitrophenyl
pNPS: PNP sulfate
AMP: Adenosine monophosphate
RBS: Ribosome-binding site
